# A novel method for efficient and abundant production of *Phytophthora brassicae *zoospores on Brussels sprout leaf discs

**DOI:** 10.1186/1471-2229-9-111

**Published:** 2009-08-22

**Authors:** Klaas Bouwmeester, Francine Govers

**Affiliations:** 1 Laboratory of Phytopathology, Wageningen University, Binnenhaven 5, 6709 PD Wageningen and Graduate School Experimental Plant Sciences, the Netherlands

## Abstract

**Background:**

*Phytophthora *species are notorious oomycete pathogens that cause diseases on a wide range of plants. Our understanding how these pathogens are able to infect their host plants will benefit greatly from information obtained from model systems representative for plant-*Phytophthora *interactions. One attractive model system is the interaction between Arabidopsis and *Phytophthora brassicae*. Under laboratory conditions, Arabidopsis can be easily infected with mycelial plugs as inoculum. In the disease cycle, however, sporangia or zoospores are the infectious propagules. Since the current *P. brassicae *zoospore isolation methods are generally regarded as inefficient, we aimed at developing an alternative method for obtaining high concentrations of *P. brassicae *zoospores.

**Results:**

*P. brassicae *isolates were tested for pathogenicity on Brussels sprout plants (*Brassica oleracea *var.* gemmifera*). Microscopic examination of leaves, stems and roots infected with a GFP-tagged transformant of *P. brassicae *clearly demonstrated the susceptibility of the various tissues. Leaf discs were cut from infected Brussels sprout leaves, transferred to microwell plates and submerged in small amounts of water. In the leaf discs the hyphae proliferated and abundant formation of zoosporangia was observed. Upon maturation the zoosporangia released zoospores in high amounts and zoospore production continued during a period of at least four weeks. The zoospores were shown to be infectious on Brussels sprouts and Arabidopsis.

**Conclusion:**

The *in vitro *leaf disc method established from *P. brassicae *infected Brussels sprout leaves facilitates convenient and high-throughput production of infectious zoospores and is thus suitable to drive small and large scale inoculation experiments. The system has the advantage that zoospores are produced continuously over a period of at least one month.

## Background

Plants can be affected by a broad range of plant-pathogenic oomycetes, such as downy mildews and *Phytophthora *species. Comprehensive knowledge of host-pathogen interactions is a prerequisite for designing novel control strategies. Elucidation of these complex interactions will especially benefit from easy and user-friendly model pathosystems. One of the potential model systems is the interaction between *Phytophthora brassicae *and Arabidopsis [1].

*P. brassicae *was initially classified as *P. porri*, a major pathogen causing white tip disease on *Allium *species [2,3], but based on detailed characterization, including isozyme pattern, ITS sequence, morphology and host-pathogenicity, it is now categorized as a new and distinct species [4,5].* P. brassicae *has a narrow host range restricted to brassicaceous plants and was shown to be pathogenic on different *Brassica *species, e.g. Chinese cabbage (*Brassica rapa *subsp. *pekinensis*), Brussels sprouts (*Brassica oleracea *var. *gemmifera*) and rutabaga (swedes) (*Brassica napus *var. *napobrassica*) [6,7]. *P. brassicae *is mostly associated with post-harvest damage that limits the marketability of cabbage heads and can reach up to 90% losses [8-10]. Although less frequently, disease symptoms have been observed on cabbage plants in the field. Colonization often starts in root or stem tissue, and subsequently progresses upwards through the vascular system, eventually colonizing the leaves. Infection and disease spread is more severe under wet weather conditions with moderate temperatures; the optimum lies between 15 and 20°C, although pathogen growth has been observed at lower temperatures down to -3°C [10].

In the last decade, Arabidopsis has become the most attractive model plant for genetic and molecular studies and consequently it is favorable as host plant for studying plant-pathogen interactions. Several oomycete pathogens have been reported to infect Arabidopsis, either naturally or under laboratory conditions. These include *Hyaloperonospora arabidopsidis*, *Albugo candida *and two *Phytophthora *species, *P. cinnamoni *and *P. brassicae *[1,11-13]. The best studied *Phytophthora *species, i.e. *P. infestans *and *P. sojae*, are incapable to infect Arabidopsis; they trigger defense responses leading to non-host resistance [14]. Roetschi et al. (2001), who first described the *P. brassicae*-Arabidopsis pathosystem, inoculated a variety of *P. brassicae *isolates on multiple Arabidopsis accessions and defence mutants, and showed that certain combinations result in compatible and others in incompatible interactions [1]. This pathosystem has the potential to become a model for studying oomycete-plant interactions, allowing concurrent molecular analysis of the host as well as the pathogen.

A disadvantage of *P. brassicae *is the fact that generating zoospores is troublesome. In nature, *Phytophthora *species produce vegetative spores, the so-called sporangia, that infect the host tissue upon germination. At lower temperatures sporangia often develop into zoosporangia that release zoospores and these then act as the infectious propagules. In the laboratory one can also use mycelium plugs or mycelial suspensions as inoculum but to mimic disease cycle in nature it is more appropriate to use sporangia or zoospores. Various laboratory protocols describe the isolation of zoospores from *in vitro *grown mycelium [15] and for several *Phytophthora *species it is relatively easy to obtain sufficient amounts of zoospores for en masse inoculation. For *P. brassicae*, however, efficient production of zoospores is not so straightforward [16]. To induce sporulation *P. brassicae *has to be cultured on soil medium  or transferred to Schmitthenner solution [15,16]. The preparation of these media is complicated and laborious and the amount of zoospores generated on these media is low. Moreover, zoospore production is dependent on pH, mycelial age and season (K. Belhaj and F. Mauch, personal communication; [16]). This study aimed at establishing a fast, simple and convenient system for production and isolation of *P. brassicae *zoospores. We first compared the pathogenicity of five *P. brassicae *isolates on Brussels sprouts (*Brassica oleracea *var.* gemmifera*) and monitored infection and colonization using bright field and fluorescence light microscopy. Subsequently, we optimized the zoospore production system. Leaf discs cut from infected Brussels sprout leaves were shown to be an excellent source for large scale production of *P. brassicae *zoospores.

## Results and Discussion

### *Phytophthora brassicae *lesion development on Brussels sprouts

**Figure 1:**
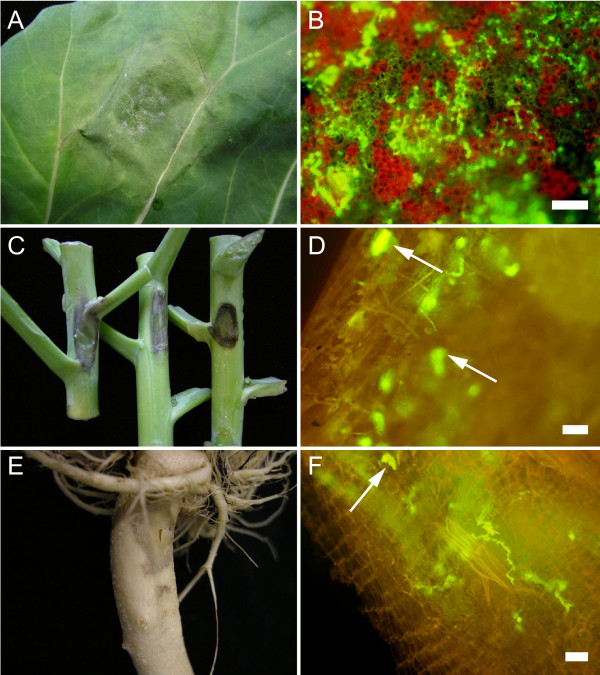
**Compatible interaction between the Brussels sprouts cultivar Cyrus and *P. brassicae***. *P. brassicae *infects leaves, stems and roots of Brussels sprouts cultivar Cyrus. Lesion development on the adaxial side of a leaf 4 days post inoculation (dpi) with isolate CBS686.95 **(A)**. Stem lesions 4 dpi with, from left to right, isolates CBS686.95, HH and GFP transformant 155m **(C)**. Root infection 4 dpi with isolate HH **(E)**. Mycelial structures visualized by GFP fluorescence in leaf **(B)**, stem **(D) **and root **(F) **tissue, 5 dpi with GFP transformant 155m. Hyphal protrusions are indicated by arrows. Scale bars represent 100 μm **(B, D) **and 10 μm **(F)**.

Mycelial plugs of *P. brassicae *were inoculated on detached leaves of Brussels sprouts cultivar Cyrus. We tested five *P. brassicae *isolates that were originally isolated from different *Brassica *crop species. Although all five were able to infect Brussels sprout leaves (Table 1), there were differences in disease progression between isolates. For example, foliar lesions caused by *P. brassicae *isolates CBS686.95 and II were predominantly larger than lesions caused by the other isolates. It is noteworthy that these two isolates were originally isolated from Brussels sprouts, possibly explaining their advantage. Foliar lesions on Brussels sprouts had a brownish-grey color and were usually surrounded by a water-soaked halo (Figure 1A). In later stages of disease development the lesion edges and especially the leaf midribs became darker in color, varying from dark-grey up to black. Another typical symptom often seen at this stage was leaf chlorosis.

**Table 1:** *P. brassicae *isolates used in this study; their origin and foliar lesion sizes on Brussels sprouts cultivar Cyrus

Isolate	Year	Country	Collected from	Lesion size ^a^
CBS178.87	1983	Germany	*Brassica rapa *subsp.*pekinensis*	2.9 ± 0.1
CBS212.82	1982	The Netherlands	*Brassica oleracea *var. *alba*	4.9 ± 0.4
CBS686.95	1995	The Netherlands	*Brassica oleracea *var.*gemmifera*	5.9 ± 0.4
HH (CBS782.97)	1994	The Netherlands	*Brassica rapa *subsp.*pekinensis*	3.5 ± 0.2
II	1994	The Netherlands	*Brassica oleracea *var.*gemmifera*	6.0 ± 0.2

^a ^the average size in cm^2 ^of at least 22 lesions at 4 dpi

The isolates were also tested for their ability to infect stems and roots (Figure 1C, and 1E). All isolates were infectious on both tissues, but – as on the leaves – there was variation in disease progression between isolates (data not shown). To better visualize the colonization process we used a Green Fluorescent Protein (GFP) tagged *P. brassicae *transformant. Microscopical examination of the infected tissues showed hyphal growth in leaves, stems and roots (Figure 1B, D, and 1F). In leaves extensive intercellular hyphal growth was found in the intercellular space between mesophyll cells. The few haustoria that were observed were small and – like haustoria of *P. infestans *– digit-like in shape. In late stages of infection, hyphae emerged through the stomata and occasionally protruded the epidermal cell layer but there was no sporulation. Instead, in leaf, stem and root lesions typical protrusions were observed (Figure 1). Supposedly, these protrusions are the sporangiophore initials. Only after being exposed to cold water sporangia were formed, which subsequently developed into zoosporangia.

### Development of a zoospore production method

**Figure 2:**
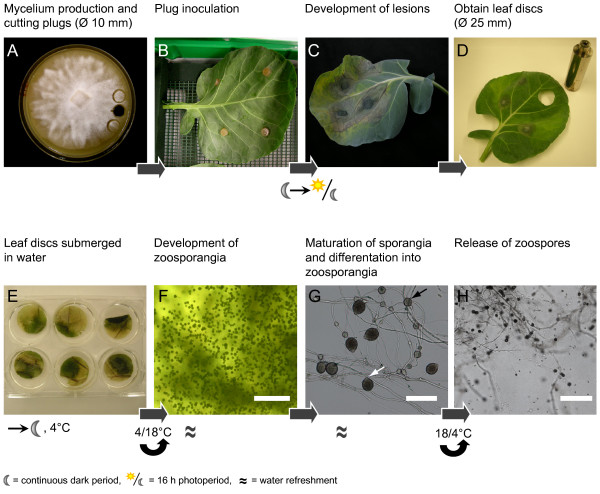
**Overview of the *P. brassicae *zoospore production procedure**. From a *P. brassicae *culture grown on V8 agar **(A) **mycelial plugs (Ø 10 mm) were cut from the actively growing margin and gently pressed on the abaxial side of Brussels sprout leaves **(B)**. From the foliar lesions **(C) **leaf discs were cut with a cork borer (Ø 25 mm) **(D) **and transferred to 6-wells plate **(E)**. The infected leaf discs were submerged in water resulting in the formation of sporangia **(F) **that developed into zoosporangia **(G) **from which zoospores are released **(H) **after being exposed to the cold for several hours. Scale bar in **(F) **and **(H) **represents 40 μm and in **(G) **100 μm. The white arrow in **(G) **points to a zoosporangium and the black arrow to a hyphal swelling.

The susceptibility of Brussels sprout leaves towards *P. brassicae *raised the idea that the lesions could be an excellent source for mass production of zoospores. Figure 2 depicts an overview of the zoospore production procedure. Inoculum was prepared by cutting mycelial plugs from *P. brassicae *colonies grown on V8 agar medium (Figure 2A). The plugs were placed on the Brussels sprout leaves (Figure 2B) with gentle pressure and with the mycelium in direct contact with the leaf surface. Lesions on the Brussels sprout leaves developed quickly and usually 4 days post inoculation (dpi) the lesions were large enough to obtain infected leaf discs with a diameter of 25 mm (Figure 2C). The leaf discs were cut with a cork borer (Ø 25 mm), placed with the abaxial side upwards in 6-well plates with 1–2 ml cold water per well and gently pushed under water (Figure 2D, E). When – after leaf disc cutting – further expansion of the foliar lesions was allowed, the infected leaf could be used to obtain new leaf discs. The first 24 hours the plates were incubated at 4°C and thereafter at 18°C. Water was refreshed with a two day interval. Infection was checked daily under a stereomicroscope. Newly formed mycelium and sporangia formation were observed after one day. After two days there was a strong increase in the number of sporangia (Figure 2F). Subsequently, the sporangia matured and developed into zoosporangia (Figure 2G). The process from appearance to maturation lasted approximately 3 days. To initiate zoospore release from mature zoosporangia fresh cold water was added and the plates were incubated at 4°C. After one hour the first zoospores were released (Figure 2H), mostly eight from each zoosporangium. The zoospores were able to swim for several hours (5 h average). A time-lapse movie showing discharged zoospores is appended (Additional file 1: Swimming *P. brassicae *zoospores).

All five isolates were tested in this system. In all cases numerous zoospores were produced and we did not observe seasonal influences. The amount of zoospores per leaf disc was semiquantitatively determined with a hemacytometer. Comparable mean numbers of zoospores per leaf disc were found for isolates CBS212.82 and II, whereas isolates HH and CBS686.95 were shown to produce more zoospores, reaching concentrations of 1*10^6 ^zoospores per ml (Table 2).

**Table 2:** Mean number of zoospores produced by a leaf disc^a^

Isolate	First harvest (zsp./ml)^b^	Second harvest (+ 8 days) (zsp./ml)
CBS212.82	1*10^5^	n.d.^c^
CBS686.95	1*10^6^	0.9 *10^5^
HH (CBS782.97)	0.5*10^6^	0.3*10^5^
II	1.5*10^5^	0.5*10^5^

^a ^Ø 25 mm, ^b ^Zsp./ml = zoospores per ml, ^c ^not determined

An additional advantage of this system is that the infected leaf discs can be reused after the first harvest. For additional zoospore harvests fresh water was added to the microwell plates every two days. Subsequently, the microwell plates were placed at 18°C to allow development and maturation of fresh zoosporangia. As in the first round, cold water was added and incubation at 4°C was used to trigger zoospore release. Zoospore yields from successive harvests were lower when compared to initial harvests (Table 2), but the concentrations were still sufficient for infection assays on plants. The leaf discs remained viable and continuously produced zoospores for a period up to one month, albeit that the concentrations became lower as the culture period proceeded.

Furthermore, in accordance with the homothallic nature of *P. brassicae*, formation of oospores was observed in the infected leaf discs, although at low frequencies and only in older leaf discs (Additional file 2: *In planta *oospore formation).

### Zoospores produced on leaf discs can infect Brussels sprouts and Arabidopsis

**Figure 3:**
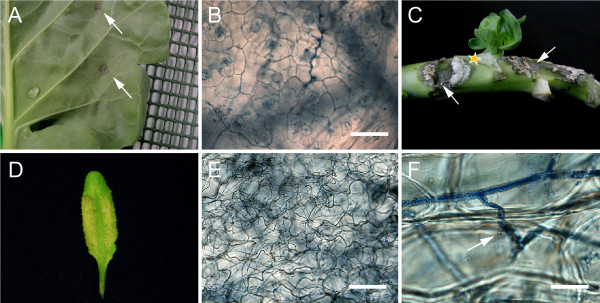
**Zoospores produced on leaf discs are infectious**. **(A) **Foliar lesions (arrows) on Brussels sprouts 4 days post inoculation (dpi) with zoospores of *P. brassicae *isolate CBS686.95. **(B) **Colonization of Brussels sprout leaf tissue. Scale bar represents 100 μm. **(C) **Infection on Brussels sprout stem tissue 3 dpi with zoospores of *P. brassicae*. Developing lesions are indicated by arrows and incidental callus formation with a yellow star. **(D) **An Arabidopsis Col-0 leaf 6 dpi with zoospores of *P. brassicae *isolate CBS686.95. **(E)**. Arabidopsis leaf colonization by intercellularly growing hyphae. Scale bar represents 100 μm. **(F)**. Intercellular hyphal growth in Arabidopsis petiole tissue. A haustorium is indicated by an arrow. Scale bar represents 20 μm. **(B, E, F) **Intercellular hyphae and haustoria were visualized by trypan blue staining.

Infectiousness of zoospores produced on leaf discs in the microwell plates was tested on Brussels sprout leaves and stems, and on Arabidopsis rosette leaves (Figure 3). The inoculations were performed as described in materials and methods. On Brussels sprout leaves, lesion development became clearly evident 2 dpi. At 4 dpi – when the lesions were remarkably larger – a typical discoloration of the tissue was observed (Figure 3A). The zoospores were also shown to infect Brussels sprout stems. Water-soaked, dark brown lesions with dense mycelial growth were observed 4 dpi (Figure 3C). Occasionally, callus formation on stem tissue was observed.

On Arabidopsis, sporulating lesions were observed at 4 dpi. Initially the lesions appeared water-soaked, thereafter the infected tissue wilted and subsequently collapsed (Figure 3D). Dried out lesions turned bleached white in color and papery in appearance. Dense tissue colonization by *P. brassicae *was observed microscopically after trypan blue staining in infected Brussels sprout and Arabidopsis tissue (Figure 3B, E, and 3F).

## Conclusion

In this report we demonstrate that *P. brassicae *easily infects Brussels sprout leaves, stems and roots. An *in vitro *leaf disc method for the isolation of *P. brassicae *zoospores was successfully established and zoospores isolated via this procedure were shown to be infectious. This method opens the opportunity to execute – on small to large scale – zoospore infections on brassicaceous plants, including Arabidopsis. The major advantages are its easy handling, the possibility of inoculating large numbers of plants and the continuous production of zoospores over a period of at least one month, at any season.

## Methods

### *P. brassicae *isolates and culture conditions

*P. brassicae *isolates used in this study were obtained from our in-house collection (i.e., II, HH) and from the Fungal Biodiversity Centre CBS, Utrecht, The Netherlands. *P. brassicae *GFP-transformant 155m [17] – which has HH as recipient background – was kindly provided by Dr. F. Mauch, University of Fribourg, Switzerland. *P. brassicae *isolates were cultured at 18°C on fresh 10% V8-juice (The Campbell Soup Co., Camden, N.J.) agar plates [15].

### Plant growth conditions

Brussels sprout plants (*Brassica oleracea *var. *gemmifera *cv. Cyrus) were grown from seed in a greenhouse in square (11 × 11 cm) plastic pots at 20–25°C, 50/70% relative humidity (RH) and a 16 h photoperiod. Experiments were conducted with 6 week old Brussels sprout plants. Arabidopsis plants were grown in special potting soil (7 parts peat: 6 parts sand: 1 part clay) in a conditioned growth chamber at 18°C with a 16 h photoperiod and at 75% RH. For inoculation 4 week old Arabidopsis plants were used.

### Infection using mycelial plugs as inoculum

Medium sized and large leaves from 6 week old Brussels sprout plants (i.e. the 6^th ^to 14^th ^leaf layer) were detached and washed with water to remove the waxy leaf surface coating. Hereafter, the leaves were placed with their petioles in water-saturated floral foam (Oasis^®^) in a tray, in such a way that the abaxial sides were facing upwards (Figure 2B). The leaves were sprayed with water and subsequently mycelial plugs (Ø 10 mm), which were taken from the margin of growing colonies, were placed firmly on the abaxial side of the leaf. The trays were closed with transparent lids, wrapped with tape to obtain high humidity, and placed in a growth chamber with a 16 h photoperiod at 18°C and a RH of 75%. The first day the trays were kept in the dark. Mycelial plugs were removed after 2–3 days to stop nutrition facilitation from the agar. Stem sections were artificially wounded with a razor blade and mycelial plugs (Ø 5 mm) were placed on the wound. The inoculated stems were incubated in the same way as the detached leaves.

### Infection using zoospores as inoculum

Leaves of Brussels sprouts (cv. Cyrus) and Arabidopsis (accession Col-0) were drop-inoculated with 10 μl droplets containing 1*10^5 ^zoospores ml^-1^. Inoculations on Arabidopsis Col-0 were conducted with the compatible *P. brassicae *isolate CBS686.95. Plants were kept at 18°C in the dark at high humidity (100% RH) for the first 24 hours after inoculation. Subsequently, plants were placed at 18°C at a relative humidity of 75–80% and a 16 h photoperiod.

### Microscopy

Fluorescence microscopy was performed with a Nikon 90i epifluorescence microscope equipped with a digital imaging system (Nikon DS-5Mc camera, Nikon NIS-AR software). GFP fluorescence was examined by using a GFP filter cube (GFP-LP, EX 460–500, DM 505, BA 510). Inoculated plant material was stained with trypan blue [18] to visualize hyphal structures and death plant cells.

## Competing interests

The authors declare that they have no competing interests.

## Authors' contributions

KB designed and performed research. KB and FG wrote the article.

## Supplementary Material

Additional file 1 **Swimming *P. brassicae *zoospores**. A time-lapse movie corresponding to figure 2H. The movie shows swimming *P. brassicae *zoospores of isolate HH. The movie lasts 3 seconds and is approximately real time. Magnification: 40×. Click here for file

Additional file 2 ***In planta *oospore formation**. An oospore of *P. brassicae *isolate II with a typical thick wall (white arrow). A black arrow points to the antheridium. The scale bar represents 50 μm. Click here for file
