# Supplementary figures and images for: A novel method for efficient and abundant production of Phytophthora brassicae zoospores on Brussels sprout leaf discs
Source: BMC Plant Biol. 2009 Aug 22;9:111. doi: 10.1186/1471-2229-9-111 (PMC2752460; doi:10.1186/1471-2229-9-111)

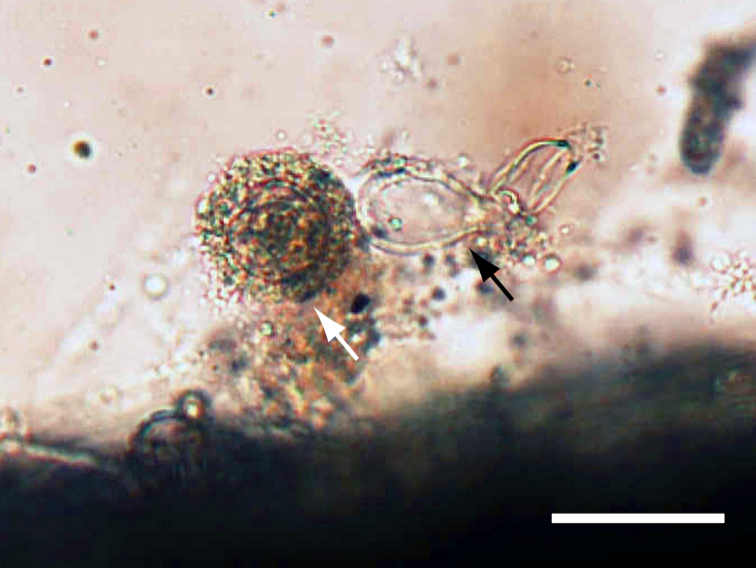

Supplement: Additional file 2 — In planta oospore formation. An oospore of P. brassicae isolate II with a typical thick wall (white arrow). A black arrow points to the antheridium. The scale bar represents 50 μm. [file 1471-2229-9-111-S2.pdf]
